# Adoption of Artificial Intelligence in the Health Care Sector

**DOI:** 10.1001/jamahealthforum.2025.5029

**Published:** 2025-11-21

**Authors:** Thuy D. Nguyen, Christopher M. Whaley, Kosali Simon, Neil Mehta, Hao Yu, Ryan K. McBain, Ateev Mehrotra, Jonathan H. Cantor

**Affiliations:** 1 Department of Health Management and Policy, University of Michigan, Ann Arbor; 2 Department of Health Services, Policy, and Practice, Brown University School of Public Health, Providence, Rhode Island; 3 O’Neill School of Public and Environmental Affairs, Indiana University, Bloomington; 4 Department of Population Medicine, Harvard Medical School and Harvard Pilgrim Health Care Institute, Boston, Massachusetts; 5 RAND Corporation, Arlington, Virginia; 6 Department of Health Services, Policy and Practice, Brown University School of Public Health, Providence, Rhode Island; 7 RAND Corporation, Santa Monica, California

## Abstract

This cross-sectional study examines artificial intelligence use in health care and other industries according to US firms participating in the 2023 to 2025 Business Trends and Outlook Survey.

## Introduction

Adoption of artificial intelligence (AI) is expected to have important implications for health care delivery and workforce.^1^ AI can shape patient education and engagement, streamline documentation, and assist clinicians with information synthesis.^2^ However, there are limited data on real-time trends of AI adoption in the health care sector and how this compares with other sectors of the economy.^3^ To fill this knowledge gap, we compared AI use in health care vs other sectors from 2023 to 2025.

## Methods

We analyzed the US Census Bureau’s Business Trends and Outlook Survey (BTOS) to examine changes in AI use from September 2023 to May 2025.^4^ In accordance with the Common Rule, this cross-sectional study was exempt from ethics review and informed consent because it used deidentified, publicly available BTOS data. We followed the STROBE reporting guideline.

The BTOS asks 1.2 million participating firms annually about their current use of AI for business purposes. Specifically, firms are asked, “In the last two weeks, did this business use … [AI] in producing goods or services? (Examples of AI: machine learning, natural language processing, virtual agents, voice recognition, etc.).” The North American Industry Classification System codes used to identify each sector and health care subsectors are described in the eMethods in Supplement 1. We reported unadjusted biweekly trends in the percentage of firms responding yes to using AI in the health care vs select other sectors.

We performed piecewise linear regression with the nl hockey program in Stata (StataCorp) to identify the break point in biweekly AI use trends. We then conducted an interrupted time series analysis to assess changes in the slope of AI use. Analyses were conducted with Stata, version 18.0. Two-sided hypothesis tests were used with a significance level of .05 (*P* < .05).

## Results

Between September 2023 and May 2025, the mean (SD) AI use in health care by firms was 5.9% (1.6%; 119 300 firm-level responses) and increased over time. In 2025, AI use in health care (8.3%) was still lower than in other sectors, such as finance and insurance (11.6%); education (15.1%); professional, scientific, and technical services (19.2%); and information services (23.2%) (Figure, A). The estimated break point in AI use trends in the health care sector was December 30, 2024, to January 12, 2025 (Figure, B; Table). At this transition, the slope shifted significantly from nearly flat in 2023 through 2024 (biweekly percentage increase: 0.005%; 95% CI, 0.004%-0.007%) to gradually increasing (0.03%; 95% CI, 0.02%-0.03%)—a 481.5% change.

**Figure.**
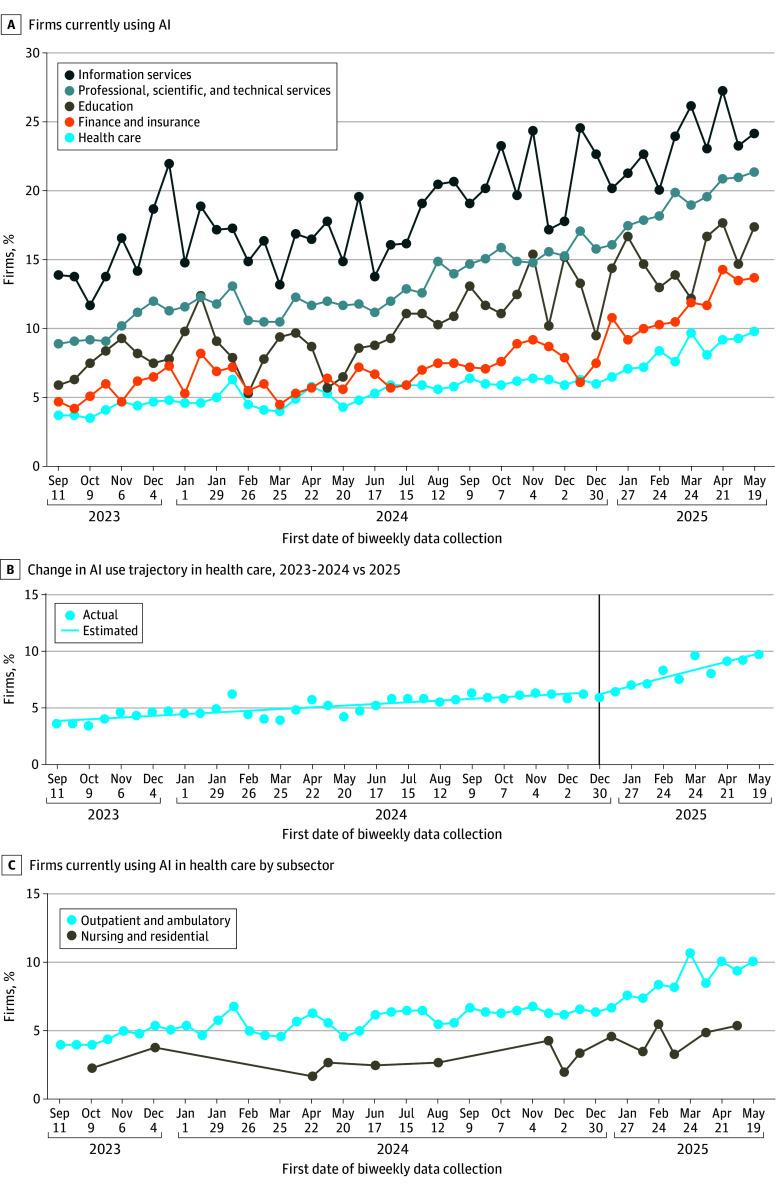
Trends in Artificial Intelligence (AI) Use in Health Care vs Non–Health Care Sectors A, The response estimate is plotted for the percentage of firms answering yes to the question: “In the last two weeks, did this business use … [AI] in producing goods or services? (Examples of AI: machine learning, natural language processing, virtual agents, voice recognition, etc.).” B, The estimated values are calculated from ordinary least squares regressions of the percentage of firms using AI on biweekly trends before and after the estimated break point on December 30, 2024. C, The US Census Bureau suppressed percentage estimates for nursing and residential care facilities for specific periods, as well as for hospitals across all periods, due to confidentiality reasons.

**Table.** Changes in Percentage of 119 300 Firm-Level Responses on Current Use of Artificial Intelligence (AI) in the Health Care Sector^a^

	Firms currently using AI, value (95% CI), %
Intercept	
Before break point^b^	6.5 (6.1-6.8)
After break point^b^	6.3 (5.7-6.9)
Change in level	−0.2
*P* value	.56
Biweekly change	
Before break point	0.005 (0.004-0.007)
After break point	0.03 (0.02-0.03)
Change in biweekly slope	0.02
*P* value	<.001
Dependent variable mean (SD)	5.9 (1.6)

^a^ The estimated means (intercepts) and biweekly change slope were calculated using ordinary least squares regression of the percentage of firms reporting current use of AI on biweekly trends.

^b^ The estimated break point was December 30, 2024, to January 12, 2025.

Within health care, the largest gains were for outpatient and ambulatory care (Figure, C), where the percentage of firms using AI increased from 4.6% in 2023 to 8.7% in 2025. Nursing and residential care facilities experienced more limited growth: 3.1% in 2023 to 4.5% in 2025.

## Discussion

Timely estimates of AI use among US firms indicate that, while AI adoption in health care lags behind other sectors, it has been rapidly increasing since 2023, particularly among outpatient and ambulatory organizations. Future research is necessary to understand the reasons and consequences of lower rate of AI adoption in health care, particularly in certain subsectors, such as nursing and residential care facilities.

The AI adoption rate observed in this study is lower than estimates in previous national studies focusing on well-resourced organizations^5^ and hospitals.^6^ For example, Poon et al^5^ examined stages of AI adoption in health care organizations from development to full deployment and found that 0% to 48% of these organizations reported full implementation among use case categories. In contrast, the BTOS tracks use of AI in producing goods and services by surveying a broader range of organizations across various sectors and employment sizes, including smaller firms. A study limitation is that BTOS subestimates for hospitals for the study period are suppressed by the US Census Bureau due to confidentiality reasons. Rapid adoption of AI in health care highlights the urgent need for active monitoring and effective regulations to ensure safe and efficient deployment of AI in patient care.

## Notes

Supplement 1. **eMethods.** Additional Details on the Business Trends and Outlook Survey Data

Supplement 2.
Data Sharing Statement
