# Supplementary material for: Adoption of Artificial Intelligence in the Health Care Sector
Source: JAMA Health Forum. 2025 Nov 21;6(11):e255029. doi: 10.1001/jamahealthforum.2025.5029 (PMC12639477; doi:10.1001/jamahealthforum.2025.5029)
Supplement: Supplement 1. — eMethods. Additional Details on the Business Trends and Outlook Survey Data [file jamahealthforum-e255029-s001.pdf]

Supplemental Online Content

Nguyen TD, Whaley CM, Simon K, et al. Adoption of artificial intelligence in the health care sector. *JAMA Health Forum*. 6(11):e255029. doi:10.1001/jamahealthforum.2025.5029

eMethods. Additional Details on the Business Trends and Outlook Survey Data

This supplementary material has been provided by the authors to give readers additional details about their work.

© 2025 Nguyen TD et al. *JAMA Health Forum*

eMethods. Additional details on the Business Trends and Outlook Survey data

The Business Trends and Outlook Survey (BTOS) is a real-time survey of the US Census Bureau intended to capture high-frequency changes in economic conditions through a qualitative survey representative of U.S. employer businesses. The BTOS sample consists of approximately 1.2 million employer businesses, drawn annually from the Business Register. The 1.2 million businesses are divided into 6 panels, with approximately 200,000 cases per panel. Businesses in each panel are asked to report up to 5 times in the collection sample year before going out of sample. They remain out of sample for at least 1 year. Data collection occurs every two weeks. Following standard Census Bureau procedures, the sample is weighted so that estimates are representative at the national, state, sector and firm size levels. BTOS data are collected via an online reporting tool. Businesses are contacted either by email or letter. A thorough description of how the questions and responses were designed can be found in a report from the Census Bureau.[1] According to this report, the 2023-2024 BTOS response rate is approximately 16%. The Census Bureau's cognitive testing indicates that most respondents understood the AI questions and were able to answer them. The testing also indicated that in situations where the respondents had a role in the company's financial or accounting services and/or were unfamiliar with the company's technical plans, they would select the correct response option "do not know".

From September 2023 onwards, BTOS data collection started covering both single and multiple location businesses; prior to that, only single location businesses were surveyed. Also starting in September 2023, BTOS included two new questions about the use of AI in the production of goods and services. These "core" AI questions are asked in reference to two time periods: current (previous two weeks) and six months in the future. The question we use in this study asks: "In the last two weeks, did this business use

[1] Bonney, Kathryn, Cory Breaux, Cathy Buffington, Emin Dinlersoz, Lucia S. Foster, Nathan Goldschlag, John C. Haltiwanger, Zachary Kroff, and Keith Savage. Tracking firm use of AI in real time: A snapshot from the Business Trends and Outlook Survey. No. w32319. National Bureau of Economic Research, 2024. http://www.nber.org/papers/w32319

© 2025 Nguyen TD et al. *JAMA Health Forum*

Artificial Intelligence (AI) in producing goods or services? (Examples of AI: machine learning, natural language processing, virtual agents, voice recognition, etc.)"

Survey results are provided for every two-week period, beginning with the period starting September 10, 2023 through the period starting May 19, 2025 (which ends by June 1, 2025). The BTOS uses the North American Industry Classification System (NAICS) to assign establishments to industries. We used the following NAICS codes to obtain the weighted estimates of current AI use at the two-digit and three-digit sector levels.

NAICS Code	Title	Description
62	Health Care and Social Assistance (the sector as a whole)	The Health Care and Social Assistance sector comprises establishments providing health care and social assistance for individuals. According to the 2017 NAICS manual, the sector includes both health care and social assistance because it is sometimes difficult to distinguish between the boundaries of these two activities. Establishments in this sector deliver services by trained professionals. All industries in the sector share this commonality of process, namely, labor inputs of health practitioners or social workers with the requisite expertise. Many of the industries in the sector are defined based on the educational degree held by the practitioners included in the industry. This sector includes four subsectors: (1) NAICS 621 – Ambulatory Health Care Services, (2) NAICS 622 – Hospitals, (3) NAICS 623 – Nursing and Residential Care Facilities, and (4) NAICS 624 – Social Assistance.
621	Ambulatory health care services	Industries in the Ambulatory Health Care Services subsector provide health care services directly or indirectly to ambulatory patients and do not usually provide inpatient services. Health practitioners in this subsector provide outpatient services, with the facilities and equipment not usually being the most significant part of the production process. This subsector comprises the following industries: (1) NAICS 6211 Offices of Physicians, (2) NAICS 6212 Offices of Dentists, (3) NAICS 6213 Offices of Other Health Practitioners, (4) NAICS 6214 Outpatient Care Centers, (5) Medical and Diagnosis Laboratories, (6) Home Health Care Services, (7) Other Ambulatory Health Care Services.

© 2025 Nguyen TD et al. *JAMA Health Forum*

622	Hospitals	Establishments in the Hospitals subsector provide inpatient health services, many of which can only be provided using the specialized facilities and equipment that form a significant and integral part of the production process. Hospitals may also provide outpatient services as a secondary activity. This subsector comprises the following industries: (1) NAICS 6221 – General Medical and Surgical Hospitals, (2) NAICS 6222 – Psychiatric and Substance Abuse Hospitals, and (3) NAICS 6223 – Specialty Hospitals (except Psychiatric and Substance Abuse).
623	Nursing and Residential Care Facilities	Industries in the Nursing and Residential Care Facilities subsector provide residential care combined with either nursing, supervisory, or other types of care as required by the residents. In this subsector, the facilities are a significant part of the production process, and the care provided is a mix of health and social services with the health services being largely some level of nursing services. This subsector comprises the following industries: (1) NAICS 6231 Nursing Care Facilities, (2) NAICS 6232 Residential Intellectual and Developmental Disability, Mental Health, and Substance Abuse Facilities, (3) NAICS 6233 Continuing Care Retirement Communities and Assisted Living Facilities for the Elderly, and (4) NAICS 6239 Other Residential Care Facilities.
624	Social Assistance	Industries in the Social Assistance subsector provide a wide variety of social assistance services directly to their clients. These services do not include residential or accommodation services, except on a short-stay basis.
51	Information	The Information sector comprises establishments engaged in the following processes: (a) producing and distributing information and cultural products, (b) providing the means to transmit or distribute these products as well as data or communications, and (c) processing data. The main components of this sector are motion picture and sound recording industries; publishing industries, including software publishing; broadcasting and content providers; telecommunications industries; computing infrastructure providers, data processing, Web hosting, and related services; and Web search portals, libraries, archives, and other information services.
52	Finance and Insurance	The Finance and Insurance sector comprises establishments primarily engaged in financial transactions (transactions involving the creation, liquidation, or change in ownership of financial assets) and/or in facilitating financial transactions. Three principal types of activities are identified:1) raising funds by taking deposits and/or issuing securities and, in the process, incurring liabilities. Establishments engaged in this activity use raised funds to acquire financial assets by making loans and/or purchasing securities; 2) pooling of risk by underwriting insurance and annuities; 3) Providing specialized services facilitating or supporting financial intermediation, insurance, and employee benefit programs.
54	Professional, Scientific, and Technical Services	The Professional, Scientific, and Technical Services sector comprises establishments that specialize in performing professional, scientific, and technical activities for others. These activities require a high degree of expertise and training. The

© 2025 Nguyen TD et al. *JAMA Health Forum*

		establishments in this sector specialize according to expertise and provide these services to clients in a variety of industries and, in some cases, to households. Activities performed include: legal advice and representation; accounting, bookkeeping, and payroll services; architectural, engineering, and specialized design services; computer services; consulting services; research services; advertising services; photographic services; translation and interpretation services; veterinary services; and other professional, scientific, and technical services.
61	Educational Services	The Educational Services sector comprises establishments that provide instruction and training in a wide variety of subjects. This instruction and training is provided by specialized establishments, such as schools, colleges, universities, and training centers. These establishments may be privately owned and operated for profit or not for profit, or they may be publicly owned and operated. They may also offer food and/or accommodation services to their students.

Notes: More details on the target population and sampling frame can be accessed at https://www.census.gov/hfp/btos/data_downloads. The full NAICS industry hierarchy and details are at https://www.census.gov/naics/reference_files_tools/2017_NAICS_Manual.pdf

© 2025 Nguyen TD et al. *JAMA Health Forum*
