# Supplementary material for: Adoption of Artificial Intelligence in the Health Care Sector
Source: JAMA Health Forum. 2025 Nov 21;6(11):e255029. doi: 10.1001/jamahealthforum.2025.5029 (PMC12639477; doi:10.1001/jamahealthforum.2025.5029)
Supplement: Supplement 2. — Data Sharing Statement [file jamahealthforum-e255029-s002.pdf]

Data Sharing Statement

Nguyen. Adoption of Artificial Intelligence in the Health Care Sector. *JAMA Health Forum*. Published November 21, 2025. doi:10.1001/jamahealthforum.2025.5029

Data
Data available: Yes
Data types: Data (not involving human participants)
How to access data: https://www.census.gov/hfp/btos/about
When available: With publication

Supporting Documents
Document types: None

Additional Information
Who can access the data: anyone requesting the data
Types of analyses: for any purpose
Mechanisms of data availability: without investigator support
